# Exploring the Action Mechanism of the Active Ingredient of Quercetin in *Ligustrum lucidum* on the Mouse Mastitis Model Based on Network Pharmacology and Molecular Biology Validation

**DOI:** 10.1155/2022/4236222

**Published:** 2022-06-10

**Authors:** Lu Cao, Tao Wang, XiaoYu Mi, Peng Ji, XingXu Zhao, Yong Zhang

**Affiliations:** ^1^College of Veterinary Medicine, Gansu Agricultural University, Lanzhou 730070, China; ^2^Provincial Key Laboratory of Animal Reproductive Physiology and Reproduction Regulation, Gansu Agricultural University, Lanzhou 730070, China

## Abstract

**Aim:**

The aim of this study is to explore the mechanism of action of quercetin, the main active anti-inflammatory component of *Ligustrum lucidum*, in the prevention and treatment of mastitis.

**Methods:**

Prediction of the main active ingredients and key anti-inflammatory targets of *Ligustrum lucidum* using a network pharmacology platform and molecular biology validation of the results. Observation of histopathological changes in the mouse mammary gland by hematoxylin-eosin staining(H&E) method, quantitative real-time PCR(qPCR), and Western blot (WB) to detect the expression levels of relevant inflammatory factors mRNA and protein.

**Results:**

A total of 7 active ingredients and 42 key targets were obtained from the network pharmacological analysis of *Ligustrum lucidum*, with quercetin as the main core ingredient and tumor necrosis factor(TNF), serine threonine protein kinase1(AKT1), and interleukin6(IL6) as the core targets; H&E results showed that pathological changes were reduced to different degrees in the dose group compared to the model group. The qPCR results showed that the relative expression of *TNF* and *IL6* mRNA in the high dose group on day 3 and the high and medium dose groups on day 7 were not significantly different compared with the blank group (*P* > 0.05), and the difference between the dose groups on day 5 was significant (*P* < 0.05). WB results showed that the difference in nuclear factor kappa-B(NF-*κ*B) protein expression in the medium and low dose groups on day 7 was significant compared with the blank group (*P* < 0.05), the difference in 5 and 7 days, significant differences in AKT1 protein expression between the middle and low dose groups (*P* < 0.05), nonsignificant differences in the TNF protein expression between the high dose groups on day 7 (*P* > 0.05), and significant differences in the IL6 protein expression between the middle and low dose groups on days 3 and 7 (*P* < 0.05).

**Conclusion:**

Quercetin, the main active ingredient of *Ligustrum lucidum*, may act in the prevention and treatment of mastitis by inhibiting the expression of inflammatory factors in phosphoinositol 3-kinase(PI3K)-AKT and NF-*κ*B signaling pathways and showa a significant dose-dependent effect. This study provides theoretical basis and clues for the control of mastitis in dairy cows.

## 1. Introduction

Mastitis in dairy cows is an inflammatory response caused by a combination of pathogenic microorganisms acting on the mammary glands of cows and is one of the most common diseases in dairy cows. In general, common environmental organisms such as *Streptococcus*, *Staphylococcus aureus*, and *Escherichia coli* are the main pathogens [1, 2]. The disease is not only widespread and highly prevalent but also has been the most damaging, costly, and difficult to control dairy disease in the global dairy farming industry [3]. Currently, the widespread use of antibiotics is still the main means to prevent and control mastitis in dairy cows, but the long-term use of large amounts of antibiotics can lead to an increase in drug-resistant strains of bacteria, and more food safety problems such as accumulation and residues of antibiotics in milk/meat products can directly or indirectly affect the health of animals and humans [4]. Therefore, it is of great significance to do a good job in the control of mastitis in dairy cows. Therefore, there is an urgent need to find a cost-effective natural medicine instead of antibiotics. Chinese herbal medicine as a natural medicine with low residue, low toxicity, and not easy to develop drug resistance [5], has not only become a hot spot for many scholars to explore nowadays but is also widely used in the clinical control of mastitis in dairy cows [6].

The fruit of the genus Ligustrum, family *Lignanaceae*, was first published in the Shen Nong Ben Cao Jing in the Eastern Han Dynasty and is known for its nourishing effects on the liver and kidney. Modern pharmacological studies have continued to show that extracts of *Ligustrum chasteum* exert anti-inflammatory, analgesic, antiviral, and immunomodulatory effects [7–9]. The use of the *Ligustrum lucidum* extract as an immunomodulator has been shown to have immunomodulatory activity on the immune system of piglets, resulting in a beneficial effect on the immune function of piglets [10]. However, the mechanism of the antibacterial and anti-inflammatory action of this drug needs further study due to the relatively complex composition of the Chinese herbal medicine such as *Ligustrum lucidum* and the large number of target sites of its extracts in the animal organism. In addition, the holistic, systematic, and comprehensive nature of network pharmacology is highly similar to the multicomponent and multitarget characteristics of traditional Chinese medicine, and the use of network pharmacology to study the molecular mechanism of action of traditional Chinese medicine has become a common research tool in recent years [11]. In this study, we used network pharmacology to analyze the active ingredients of *Ligustrum lucidum*, identify the active ingredients, and design the pathway to validate them experimentally by predicting the relevant mechanisms, and investigate the mechanism of action of the main active ingredients on mastitis model in mice using H&E, qPCR, and Western blot to provide a theoretical basis for later research on mastitis prevention and control in cows.

## 2. Materials and Methods

### 2.1. Screening of the Active Ingredients of *Ligustrum lucidum* and Target Acquisition

In the TCMSP (Traditional Chinese Medicine System Pharmacology Database and Analysis Platform) (http://tcmspw.com), two parameters of oral availability (OB) ≥ 30% and drug-like properties (DL) ≥ 0.18 were set for the preliminary screening of potential active ingredients of *Ligustrum lucidum*, while the target proteins corresponding to these potential active compounds were obtained using this database, de-duplicated and then passed through the UniProt protein database (https://www.uniprot.org) for correction, and the corrected target proteins were transformed into the corresponding gene names.

### 2.2. Acquisition of Anti-Inflammatory Targets

Inflammation-related disease genes were obtained by searching the GeneCards database (https://www.genecards.org) using the keyword “anti-inflammation”. The collected disease targets were aggregated and the redundant duplicate targets were removed to obtain the relevant predicted anti-inflammatory targets.

### 2.3. Visual Analysis and Network Construction of Anti-Inflammatory Targets of *Ligustrum lucidum*

The intersecting genes (key target genes) of *Ligustrum lucidum* constituent target genes and inflammatory target genes were obtained by bioinformatics online mapping to draw a Venn diagram. The screened *Ligustrum lucidum* chemical components and predicted anti-inflammatory targets were imported into Cytoscape 3.7.1 software to construct a herbal-active-component-predicted-target visual network map.

### 2.4. Construction of the Protein Interaction Network

The intersecting genes obtained after matching *Ligustrum lucidum* with anti-inflammatory target genes were imported into the String database (https://string-db.org) under “Multiple proteins”, with the species restricted to “*Bos taurus*”. Under “multiple proteins”, the species was limited to “*Bos taurus*”, and the PPI network was constructed by using the String database to collect the relevant predicted protein-protein interactions (PPI), and the PPI network was constructed for the common targets of active ingredients and inflammation in *Ligustrum lucidum*. The minimum required interaction score was selected as 0.400, and “Send network to Cytoscape” was selected in “Exports” to link to Cytoscape 3.7.1. The “Analyze Network” tool was used to analyze the topology of the network, draw a network map, and filter the core targets.

### 2.5. Animal Experiment

#### 2.5.1. Laboratory Animals, Strains, and Drugs

46 clean-grade female rats and 23 male rats of 7–8 weeks old were purchased from the Laboratory Animal Center of Lanzhou University. They were fed with standard feed and free drinking water.

*Staphylococcus aureus* was derived from milk samples of cows suffering from mastitis in Wuzhong area of Ningxia. It was isolated and identified by our laboratory and stored at −80°C. Quercetin, sourced from Nanjing Yuanzhi Biotechnology Co. Ltd. purity ≧ 98%, batch number: Yz051621, was stored at −20°C and protected from light.

#### 2.5.2. Apparatus and Reagents

A real-time fluorescent quantitative PCR instrument, gel imaging system, electrophoresis instrument, electrotransformer (Bio-Rad, USA), nucleic acid micrometer (PULTTON, USA), and ultrasensitive multifunctional imager (GE, USA) were used to conduct the research.

TRIzol total RNA extraction reagent, Thermo Scientific RevertAid First Strand cDNA Synthesis Kit, TransStart Top Green qPCR Super Mix, Soleibao MarkerIDNA Ladder, Soleibao Rainbow 180 Broad-spectrum Protein Marker (11-180KD), RIPA Lysis Solution, Soleibao 4 × protein loading buffer, white shark ECL ultrasensitive luminescent solution, etc. were used. PCR primers are synthesized by Xi'an Shenggong Biological Engineering Co. Ltd. and other reagents are domestically pure.

Rabbit-derived tumor necrosis factor (TNF), interleukin6 (IL6), serine/threonine protein kinase (AKT), transcription factor NF-*κ*B (NF-kappa-B, NF-*κ*B) antibody, and Goat Anti-rabbit IgG HRP secondary antibody (Boaosen, Beijing, China).

#### 2.5.3. Experimental Grouping and Establishment of the Drug Model

The concentration of *Staphylococcus aureus* was calculated by the plate counting method, and the optimal concentration was selected for mouse attack pre-test, and the optimal concentration of inflammatory bacteria for a lactating mouse was 1.9 × 10^5^ CFU/mL. After the mice were sexually mature, they were caged in a 1 : 2 ratio of female:male and then came into heat at the same time. The female rats about 10 days after pups were randomly divided into three time periods, day 3, day 5 and day 7, and each time period was divided into 5 groups of 3 rats each, totaling 45 rats. They were the normal group, model group, quercetin high dose group (150 mg/kg), quercetin medium dose group (100 mg/kg), and quercetin low dose group (50 mg/kg), respectively. Related studies have demonstrated that the oral LD50 of quercetin is about 160 mg/kg [12].The rabbits showed no symptoms when the dose of quercetin was 100–150 mg/kg by intravenous injection [13].Therefore, the above three groups of drug doses were selected. The mice in the normal group were injected with 50 *μ*L of saline intradermally in the fourth pair of mammary parts, and the mice in the remaining groups were injected with 50 *μ*L of the optimum concentration of *S. aureus* at the same sites. 24 hours later, the normal and model groups were instilled with equal volumes of saline each day, and the drug group was calculated by 0.2 mL/10 g and the body mass of the mice weighed before each administration. The drug was administered at 9 : 00 each day, and all 5 groups of mice were dissected at each time period until all were completed on day 7.

#### 2.5.4. H&E Staining

Mice were dissected and the more intact side of the fourth pair of mouse mammary tissue was taken and fixed using formaldehyde solution for 24 hours. Xylene dewaxing, alcohol gradient dehydration, hematoxylin staining, 1% hydrochloric acid alcohol fractionation, eosin staining, alcohol gradient dehydration, xylene transparency, and neutral gum sealing. Histopathological changes were observed under the light microscope.

#### 2.5.5. Real-Time Fluorescent Quantitative PCR (qPCR)

The relative expression levels of TNF and IL6 inflammatory factors were measured by real-time fluorescence quantitative PCR with GAPDH as the reference gene. The total RNA was extracted by TRIzol method. After extraction, 1 *μ*L was taken in an ultramicro nucleic acid protein detector to determine the concentration and OD value of RNA (A260 nm/A280 nm at 1.8∼2.0). According to the results, the RNA concentration was adjusted to 300 ng/*μ*L. cDNA was synthesized by the three-step method according to the instructions of RevertAid First Strand Kit, and stored at −20°C.

PCR reactions were performed using cDNA as the template. The total reaction system was 20 *μ*L: 2×TransStart Top Green qPCR Super Mix10 *μ*L, 0.5 *μ*L each of upstream and downstream primers, ddH_2_O_8_ *μ*L, cDNA1 *μ*L. The reaction procedure was a three-step method: 95°C 300 s, 95°C 30 s, 57°C 34 s, 72°C 25 s, 45 cycles, and it was repeated thrice for each sample. After the amplification reactions were completed, the results were data processed using the 2^−ΔΔCt^ method to calculate the relative expressions of *TNF*, *IL6,* and *GAPDH*. The primer sequences are shown in Table 1.

#### 2.5.6. Western Blot

Take about 100 mg of the mouse mammary gland tissue, add liquid nitrogen, and grind it into a powder form. Add 1 mL of high efficiency RIPA lysis solution and 10 *μ*L of PMSF, mix well, then lyse at 4°C for 1 h (vortex mixing every ten minutes), centrifuge at 4°C for 20 min at 1000 r, and aspirate the supernatant. The supernatant was aspirated. The protein was denatured at 95°C for 5 min at a ratio of 1 : 3 V_SDS loading buffer_:V _protein_ and stored at −20°C. After preparation of 12 % separation gel and 5 % SDS-PAGE concentrated gel for electrophoresis, membrane transfer, 5 % skimmed milk powder closure for 2 h, incubate primary antibody GAPDH (1 : 3000), TNF (1 : 300), IL6 (1 : 300), AKT (1 : 300), NF-*κ*B (1 : 300) at 4°C overnight, wash the membrane with PBST for 45 min and then incubate a secondary antibody (1 : 5000) was incubated in a shaker at 37°C for 2 h, and the membrane was washed with PBST for 45 min. A ECL chemiluminescence reagent was developed, and the grayscale values were scanned by Image-Pro Plus 6.0 software.

### 2.6. Statistical Analysis

The data were analyzed using SPSS 26.0 and expressed as “mean ± standard deviation” (x ± s). One-way ANOVA was used to compare the means of multiple groups, and the LSD-t test was used for two-way comparison between groups. *P* < 0.05 means the difference is significant, and *P* < 0.01 means the difference is highly significant.

## 3. Results

### 3.1. Visualization of the Main Active Ingredients of *Ligustrum lucidum* and Core Anti-Inflammatory Targets

The TCMSP database was searched to obtain 13 compounds of *Ligustrum lucidum*, 7 of which were active ingredients. The corresponding target proteins of the potential active ingredients were obtained using the TCMSP database, and the predicted target protein names were converted to gene names by the Uniprot database. The GeneCards database was searched to obtain the intersection with *Ligustrum lucidum* target genes, and 42 common targets were obtained. The common target proteins and the seven active ingredients of *Ligustrum lucidum* were imported into Cytoscape 3.7.1 software to construct *Ligustrum lucidum* active ingredient-predicted target network (Supplementary Figure 1). The blue circular nodes are the common targets of drug and inflammation, and the red diamonds are the active ingredients of *Ligustrum lucidum* that play a major role. The largest degree value (darkest color, highest number of nodes) is quercetin, indicating that quercetin is the most important active component of *Ligustrum lucidum* exerting anti-inflammatory effects.

### 3.2. PPI Network Construction Analysis

The 42 common target proteins obtained above were imported into the String 11.0 database to construct the PPI network of *Ligustrum lucidum* and inflammation (Supplementary Figure 2), which contained 42 nodes and 379 edges with an average node (key target) degree value of 18. The degree value was positively correlated with the node size and color shade. The thickness of the node-to-node line indicates the size of the combined score, and the thicker the line, the larger the value, the stronger the interaction. The core targets with degree>20 were selected according to the degree value, and the top 3 were TNF, AKT1, and IL6.

### 3.3. Effect of Quercetin on *Staphylococcus* a*ureus*-Induced Mouse Mastitis Model

#### 3.3.1. Histopathological Changes of Mouse Mammary Glands

The H&E results showed (Supplementary Figure 3) that the blank group on days 3, 5, and 7 had intact glandular vesicle structure, no edema, or hyperplasia in the glandular vesicle wall, and significant lipid droplets in the glandular vesicle lumen. In the three stage model group, it was obvious that the glandular follicle structure was severely damaged, the glandular lumen was filled with shed epithelial cells, and there were a large number of lymphocytes as well as bruises in the glandular interstitial space. In the high-dose group, the glandular follicle structure was intact but slightly congested, and there was a reduction in the area of milk staining in the glandular follicle, with no significant change in comparison between 3, 5, and 7 days, and overall convergence to the normal group state. In the middle dose group, there was hyperplasia in the wall of the glandular follicle compared with the high dose group, and there was a small amount of inflammatory cells in the lumen of the glandular follicle, and the congestion was reduced on days 5 and 7 compared with day 3. In the low-dose group, the glandular follicle structure was incomplete, with edema and hyperplasia, congestion, and bruising, but it was reduced to some extent compared with the model group. The abovementioned results indicated that the pathological model of mastitis in mice was successfully established and quercetin had a significant protective effect on *S. aureus*-induced mastitis in mice.

#### 3.3.2. Detection of the Relative Expression of TNF and IL6 mRNA in the Mouse Breast Tissue by qPCR

As shown in Supplementary Figure 4, the relative expression of TNF and IL6 mRNA was significantly lower in each dose group on days 3, 5, and 7 after treatment compared with the model group (*P* < 0.05).

At day 3 of treatment, the differences in the relative expression of TNF and IL6 mRNA between the dose groups were significant (*P* < 0.05); at day 5 of treatment, the differences in the relative expression of TNF mRNA between the dose groups were significant (*P* < 0.05), and the differences in the relative expression of IL6 mRNA between the high and medium dose groups were not significant (*P* > 0.05); at day 7 of treatment, the differences in the relative expression of TNF and IL6 mRNA relative expression in the high and medium dose groups were not significantly (*P* > 0.05), and the differences between each dose group compared with the low dose were significant (*P* < 0.05).

#### 3.3.3. Detection of NF-*κ*B, AKT1, TNF, and IL6 Protein Expression in Mouse Breast Tissues by WB

As shown in Supplementary Figure 5, NF-*κ*B protein expression, compared with the model group, was significantly different (*P* < 0.05) between the model and dose groups on days 3 and 5, except for the medium and low dose groups on day 7, where the difference was not significant (*P* > 0.05). The differences were not significant (*P* > 0.05) on day 3 and 5 and significant (*P* < 0.05) on day 7 compared to each dose group.

AKT1 protein expression, compared with the model group, differed significantly (*P* < 0.05) between the model and dose groups on days 3 and 5, except for the low dose group on day 7, where the difference was not significant (*P* > 0.05). The differences were not significant (*P* > 0.05) on day 3 and significant (*P* < 0.05) on days 5 and 7 compared to each dose group.

TNF protein expression, compared with the model group, differed significantly (*P* < 0.05) between the model and dose groups on days 3 and 5, except for the low dose group on day 7, where the difference was not significant (*P* > 0.05). The differences were not significant (*P* > 0.05) on day 3 and significant (*P* < 0.05) on days 5 and 7 compared to each dose group.

IL6 protein expression, compared to the model group, differed significantly (*P* < 0.05) among the dose groups at all time periods. The differences were not significant (*P* > 0.05) on days 3 and 5 and significant (*P* < 0.05) on day 7 compared to each dose group.

## 4. Discussion

Cows with acute clinical mastitis have typical symptoms such as swollen and red udder to the eye, hot and painful to palpation, and the presence of hard lumps. The main pathological changes are an increased number of leukocytes in the blood, the presence of blood-like milk, clots or flocculent material in the milk, and a decrease in milk production [14]. Some studies have shown that the main causative factor of mastitis in dairy cows is infection by pathogenic microorganisms, and *S. aureus* is one of the most important and common pathogens that induce the disease [15]. *S. aureus* can repeatedly infect the mammary tissue of cows, inducing an inflammatory response in the udder, apoptosis, and the release of exotoxins that can cause systemic symptoms and eventually lead to the death of the cow [16].It also causes occult mastitis, which is not clinically evident and lasts for a long time, causing great losses to the dairy industry [17]. Nowadays, the misuse of antibiotics causing excessive drug residues in milk and the development of microbial resistance is one of the most serious and urgent problems in dairy farming. In order to reduce the harm caused by antibiotic treatment of mastitis in dairy cows, Chinese medicines are yet to be developed as new drugs for the treatment of this disease.

In this study, the main active components of *Ligustrum lucidum* and their anti-inflammatory effects were elucidated from multiple perspectives by linking “drug-target-disease” systematically by means of network pharmacological analysis. The predicted results suggest that quercetin has potential anti-inflammatory effects. The literature shows that products containing natural quercetin have a strong protective effect against mastitis in cows. In addition to acting as a bactericide, it also inhibited LPS, *Escherichia coli,* and *Staphylococcus aureus*-induced apoptosis of mammary epithelial cells in cows [18].In addition, the core targets obtained were TNF, IL6, and AKT1. It was found that induction of inflammation in human retinal pigment epithelial cells with TNF and intervention with quercetin could inhibit the expression of related proteins [19]; in the treatment of inflammatory processes in cardiovascular diseases, quercetin has a C3-OH moiety that inhibits LPS-stimulated cytokines, thus significantly reducing the protein and mRNA expression, with potential therapeutic effects on the inflammatory process in cardiovascular diseases [20]; It has also been predicted by relevant network pharmacology that the active ingredient quercetin, a drug for knee osteoarthritis, is well matched to the AKT1 target [21]; however, the effect of Si-Miao-San on knee osteoarthritis and its potential mechanisms have not been evaluated. Therefore, the results of the abovementioned study are similar in accuracy to those predicted by networked pharmacology in this study. The difference lies in the fact that this study used networked relationships to regulate the relevant components and factors as a whole, providing a theoretical basis for later experimental validation and exploring the mechanism of action of *Ligustrum lucidum* against mastitis in dairy cows, as well as providing a new approach and a new idea for the application of traditional Chinese medicine to mastitis in dairy cows.

From the pathological histological point of view, it was observed that the mammary epithelial cells in the blank control mice were structurally intact and tightly connected. In contrast, the mammary epithelial cells in the model group were degenerative and necrotic, with a large number of inflammatory cells infiltrating, and the glandular vesicle structure was destroyed, with erythrocyte exudation and aggregation in the interstitium. These phenomena are consistent with the results of Wang Yong sheng and Zhang Zhen Biao et al. who established a mouse mastitis model [22, 23], indicating that the experiment successfully constructed a mouse mammary gland model. After different doses of quercetin intervention, the mammary epithelial cells in the high-dose group were significantly restored compared with the middle and low-dose groups, but a small amount of red blood cells were still exuded, indicating that quercetin could repair damaged epithelial cells and thus achieve the purpose of restoring glandular follicle function. An experimental study found that quercetin significantly inhibited the degree of ankle swelling in rats with gouty arthritis and attenuated the histological signs of acute inflammation in treated animals, indicating that quercetin has a better anti-inflammatory and analgesic effect [24]. Similarly, a study investigated the antibacterial activity of quercetin using the drug-sensitive paper method and found that it was most effective against *Bacillus subtilis* and *Escherichia coli* [25]. The present experimental H&E results are in agreement with the abovementioned studies, indicating that quercetin, the main active ingredient of *Ligustrum lucidum*, has significant anti-inflammatory effects.

To further validate the anti-inflammatory effect of quercetin, the main active component of *Ligustrum lucidum*, on mastitis in dairy cows as derived from network pharmacological analysis, the expression of TNF and IL6 mRNA was detected using qPCR. TNF and IL6 are involved in a variety of cell biological effects and are typical pro-inflammatory cytokines [26]. Also, TNF and IL6 mediate the onset and development of chronic inflammation in the body [27, 28] and therefore can be used as important indicators to assess the inflammatory response. The results of this study showed that the mRNA expressions of *TNF* and *IL6* in the model group on days 3, 5, and 7 were significantly higher than those in the blank group and the treatment group, indicating that *S. aureus* at a concentration of 1.9 × 10^5^ CFU/mL exerted the most desirable pro-inflammatory effect after acting on the mouse udder. Since the mRNA expressions of *TNF* and *IL6* in the model group on day 7 were much higher than those on days 5 and 3, the reason for this phenomenon might be the conversion of the organism from chronic inflammation to acute inflammation, but the exact mechanism remains to be verified. Compared with the model group, both *TNF* and *IL6* mRNA expression were significantly lower in the treatment group, thus indicating that quercetin, the main active component of *Ligustrum lucidum*, played a significant role in suppressing inflammation. A network pharmacology analysis identified two key targets of the formula for strengthening the spleen, dispelling dampness, and resolving blood stasis, mainly including quercetin and kaempferol, and an experiment confirmed that the formula could exert anti-chronic glomerulonephritis effects by reducing TNF and IL6 mRNA expression [29]. This result is consistent with the above results. Comparison between dose groups revealed that TNF and IL6 mRNA expressions were positively correlated with decreasing quercetin concentrations in all dose groups except IL6 on day 3. Therefore, quercetin, the main active ingredient of *Ligustrum lucidum*, was found to be protective against mastitis in mice in a dose-dependent manner. By comparing between different time periods, it was found that the best treatment effect was achieved at day 5 for high, medium, and low doses.

To investigate the ability of quercetin, the main active ingredient of *Ligustrum lucidum*, to inhibit the development of mastitis in mice and its mechanism of action. In this experiment, the expression of proteins related to PI3K-AKT and NF-*κ*B signaling pathways were analyzed by Western blot. The results showed that quercetin was able to reduce the protein expression levels of NF-*κ*B, AKT1, TNF, and IL6. AKT is a serine/threonine protein kinase that has been classified into three isoforms (AKT1, AKT2, and AKT3) based on differences in its residues, with AKT1 being more commonly expressed. In the PI3K-AKT signaling pathway, AKT acts as a key protein and the activation of this pathway can cause a series of cellular life activities [30, 31].It was found that the PI3K-AKT signaling pathway is thought to play an important role in the inflammatory response by regulating NF-*κ*B [32]. It has been probed that infection of mammary epithelial cells by *Streptococcus tuberculosis* can activate the interaction between PI3K-AKT signaling pathway and NF-*κ*B signaling pathway thereby promoting inflammation [33]. The NF-*κ*B signaling pathway is associated with the transcription of many inflammatory genes and is also a central hub of the TNF signaling pathway that can mediate the release of several downstream inflammatory factors, such as TNF and IL6 [34]. Related experiments have verified that rutin inhibits LPS-induced mastitis in mice through NF-*κ*B signaling [35].This study concluded that the protein expression in the *S. aureus*-induced mastitis model group of mice was significantly higher than that in the blank group. The expression of NF-*κ*B, AKT1, TNF, and IL6 in the mammary gland tissues of mice decreased significantly with the increase of quercetin dose in the treatment group, indicating that quercetin inhibited the process of *S. aureus*-induced inflammation in the mammary gland of mice, and its effect was significantly dose-dependent, and the effect was most obvious at high doses. In a related study, different concentrations of quercetin were applied to a rat model of cerebral hemorrhage, resulting in a significant reduction in the symptoms of cerebral hemorrhage in the high-dose group, a decrease in the expression of related factors and proteins and a protective effect on neuronal cells [36]; it is also consistent with the findings of Xiong G [37] and others. This suggests that quercetin can inhibit bacterial attack on cow udder and provide significant protection against it. Meanwhile, the results of the study revealed that the reduced expression of NF-*κ*B, AKT1, TNF, and IL6 proteins may be related to the involvement of PI3K-AKT and NF-*κ*B signaling pathways in the development of mastitis in mice, thus exerting anti-inflammatory effects in a significant dose-dependent manner. The difference is that this study further clarifies the issue of drug regimen and verifies its therapeutic mechanism based on solving the drug dosage, making the drug more accurate and practical for clinical application.

## 5. Conclusion

In summary, network pharmacology successfully predicted the main anti-inflammatory active components and the key targets of action of *Ligustrum lucidum*. The results of in vivo tests indicated that the anti-inflammatory effect possessed by quercetin may be related to the inhibition of PI3K-AKT and NF-*κ*B signaling pathway activity by reducing the expression of inflammatory factors. This confirms that quercetin is promising as a potential candidate for the treatment and prevention of mastitis in dairy cows. It provides an experimental basis for the clinical application of quercetin to prevent and treat mastitis in dairy cows.

## Figures and Tables

**Table 1:** Information of the primer sequences.

Gene	Gene number	Primer sequences	Product length	Tm (°C)
*GAPDH*	NM_001289726.1	F : GGAGCGAGACCCCACTAACAT R : TAAGGGGGCTAAGCAGTTGGT	247	57
*TNF*	NM_013693.3	F : AAACCACCAAGTGGAGGAGC R : ACAAGGTACAACCCATCGGC	120	60
*IL6*	NM_031168.2	F : GTCCTTCCTACCCCAATTTCCA R : TAACGCACTAGGTTTGCCGA	156	57

## Data Availability

The data used to support the findings of this study are included within the article and Supplementary Materials file.

## Supplementary Materials

Supplementary Materials Supplementary MaterialsFigure 1: “TCM - active ingredient - Anti-inflammation target” network of forsythia. Figure 2: PPI network of common targets of forsythia and Anti-inflammation. Figure 3: Mouse breast tissues H&E staining(40 ×). Figure 4: Relative expression of TNF and IL6 mRNA at different time periods. Different lowercase letters indicate significant differences (*P* < 0.05); the same lowercase letters indicate not significant differences (*P* > 0.05); the same below. Figure 5: Expression of NF-*κ*B, AKT1, TNF and IL6 proteins at different time periods.

## References

[1] Ganaie M. Y., Qureshi S., Kashoo Z. (2018). Isolation and characterization of two lytic bacteriophages against *Staphylococcus aureus* from India: newer therapeutic agents against Bovine mastitis. *Veterinary Research Communications*.

[2] Wang D., Zhang L., Yong C. (2017). Relationships among superantigen toxin gene profiles, genotypes, and pathogenic characteristics of *Staphylococcus aureus* isolates from bovine mastitis. *Journal of Dairy Science*.

[3] Melchior M. B., Vaarkamp H., Fink-Gremmels J. (2006). Biofilms: a role in recurrent mastitis infections?. *The Veterinary Journal*.

[4] Santman-Berends I. M. G. A., Gonggrijp M. A., Hage J. J. (2017). Prevalence and risk factors for extended-spectrum beta-lactamase or AmpC-producing *Escherichia coli* in organic dairy herds in The Netherlands. *Journal of Dairy Science*.

[5] Mushtaq S., Shah A. M., Shah A. (2018). Bovine mastitis: an appraisal of its alternative herbal cure. *Microbial Pathogenesis*.

[6] Shang F., Li L., Yu L., Ni J., Chen X., Xue T. (2019). Effects of stigmata maydis on the methicillin resistant Staphylococusaureus biofilm formation. *PeerJ*.

[7] Wu C. R., Lin W. H., Lin Y. T., Wen C. L., Ching H., Lin L. W. (2011). Analgesic and anti-inflammatory property of the methanol extract from ligustrum morrisonense leaves in rodents. *The American Journal of Chinese Medicine*.

[8] Hu X. P., Shao M. M., Song X. (2016). Anti-influenza virus effects of crude phenylethanoid glycosides isolated from ligustrum purpurascens via inducing endogenous interferon-*γ*. *Journal of Ethnopharmacology*.

[9] Song X., Li C. Y., Zeng Y. (2012). Immunomodulatory effects of crude phenylethanoid glycosides from Ligustrum purpurascens. *Journal of Ethnopharmacology*.

[10] Wang J., Shan A., Liu T., Zhang C., Zhang Z. (2012). In vitro immunomodulatory effects of an oleanolic acid-enriched extract of Ligustrum lucidum fruit (Ligustrum lucidum supercritical CO2 extract) on piglet immunocytes. *International Immunopharmacology*.

[11] Wang X., Wang Z. Y., Zheng J. H., Li S. (2021). TCM network pharmacology: a new trend towards combining computational, experimental and clinical approaches. *Chinese Journal of Natural Medicines*.

[12] Sullivan M., Follis R. H., Hilgartner M. (1951). Toxicology of podophyllin. *Proceedings of the Society for Experimental Biology and Medicine*.

[13] Bjeldanes L. F., Chang G. W. (1977). Mutagenic activity of quercetin and related compounds. *Science*.

[14] Bianchi R. M., Schwertz C. I., de Cecco B. S. (2019). Pathological and microbiological characterization of mastitis in dairy cows. *Tropical Animal Health and Production*.

[15] Wang M., Zhang Y., Zhu J. (2016). Anti-Staphylococcus aureus single-chain variable region fragments provide protection against mastitis in mice. *Applied Microbiology and Biotechnology*.

[16] Bannerman D. D., Paape M. J., Lee J. W., Zhao X., Hope J. C., Rainard P. (2004). *Escherichia coli* and *Staphylococcus aureus* elicit differential innate immune responses following intramammary infection. *Clinical and Diagnostic Laboratory Immunology*.

[17] Ebrahimie E., Mohammadi-Dehcheshmeh M., Laven R., Perovski K. R. (2021). Rule discovery in milk content towards mastitis diagnosis:dealing with farm heterogeneity over multiple years through classification based on associations. *Animals*.

[18] Wang K., Jin X. L., Shen X. G. (2016). Effects of Chinese propolis in protecting bovine mammary epithelial cells against mastitis pathogens-induced cell damage. *Mediators of Inflammation*.

[19] Cheng S. C., Wu Y. H., Huang W. C., Pang J. H. S., Huang T. H., Cheng C. Y. (2019). Anti-inflammatory property of quercetin through downregulation of ICAM-1 and MMP-9 in TNF-*α*-activated retinal pigment epithelial cells. *Cytokine*.

[20] Zaragozá C., Villaescusa L., Monserrat J., Zaragozá F., Álvarez-Mon M. (2020). Potential therapeutic anti-inflammatory and immunomodulatory effects of dihydroflavones Flavones, and Flavonols. *Molecules*.

[21] Huang Z., Shi X., Li X. (2020). Network pharmacology approach to uncover the mechanism governing the effect of simiao powder on knee osteoarthritis. *BioMed Research International*.

[22] Wang Y. S., Teng G. Q., Zhou H., Dong C. l. (2020). Germanium reduces inflammatory damage in mammary glands during lipopolysaccharide-induced mastitis in mice. *Biological Trace Element Research*.

[23] Zhang Z. B., Guo Y. F., Li C. Y., Qiu Cw., Guo My. (2019). Selenium influences mmu-miR-155 to inhibit inflammation in*Staphylococcus aureus*-induced mastitis in mice. *Food & Function*.

[24] Huang J., Zhu M., Tao Y. (2012). Therapeutic properties of quercetin on monosodium urate crystal-induced inflammation in rat. *Journal of Pharmacy and Pharmacology*.

[25] Sonibare M. A., Aremu O. T., Okorie P. N. (2016). Antioxidant and antimicrobial activities of solvent fractions of Vernonia cinerea (L.) Less leaf extract. *African Health Sciences*.

[26] Cheng X. Y., Li J. L. (2015). Biological activity of cytotoxic dendritic cells cocultured with cytokine-induced killer cells and their effect on acute leukemia cells. *Genetics and Molecular Research*.

[27] Batra R., Suh M. K., Carson J. S. (2018). IL-1*β* (Interleukin-1*β*) and TNF-*α* (tumor necrosis factor-*α*) impact abdominal aortic aneurysm formation by differential effects on macrophage polarization. *Arteriosclerosis, Thrombosis, and Vascular Biology*.

[28] Mantovani A., Cassatella M. A., Costantini C., Jaillon S. (2011). Neutrophils in the activation and regulation of innate and adaptive immunity. *Nature Reviews Immunology*.

[29] Liu T., Gao Y. C., Qin X. J., Gao J. R. (2021). Exploring the mechanism of Jianpi Qushi Huayu Formula in the treatment of chronic glomerulonephritis based on network pharmacology. *Naunyn-Schmiedeberg’s Archives of Pharmacology*.

[30] Gajewska M., Zielniok K., Debski B., Motyl T. (2013). IGF-I retards proper development of acinar structures formed by bovine mammary epithelial cells via sustained activation of Akt kinase. *Domestic Animal Endocrinology*.

[31] Lu X. L., Zhao C. H., Yao X. L., Zhang H. (2017). Quercetin attenuates high fructose feeding-induced atherosclerosis by suppressing inflammation and apoptosis via ROS-regulated PI3K/AKT signaling pathway. *Biomedicine & Pharmacotherapy*.

[32] Gao F., Gao E., Yue T. L. (2002). Nitric oxide mediates the antiapoptotic effect of insulin in myocardial ischemia-reperfusion. *Circulation*.

[33] Su S., Li X., Li S. (2019). Rutin protects against lipopolysaccharide-induced mastitis by inhibiting the activation of the NF-*κ*B signaling pathway and attenuating endoplasmic reticulum stress. *Inflammopharmacology*.

[34] Freitas R. H., Fraga C. A. (2018). NF-*κ*B-IKK*β* pathway as a target for drug development: realities, challenges and perspectives. *Current Drug Targets*.

[35] Li B., Xi P., Wang Z. (2018). PI3K/Akt/mTOR signaling pathway participates in Streptococcus uberisinduced inflammation in mammary epithelial cells in concert with the classical TLRs/NF-ĸB pathway. *Veterinary Microbiology*.

[36] Zhang Y. F., Yi B., Ma J. H. (2015). Quercetin promotes neuronal and behavioral recovery by suppressing inflammatory response and apoptosis in a rat model of intracerebral hemorrhage. *Neurochemical Research*.

[37] Xiong G., Ji W., Wang F. (2019). Quercetin inhibits inflammatory response induced by LPS from porphyromonas gingivalis in human gingival fibroblasts via suppressing NF-*κ*B signaling pathway. *BioMed Research International*.

