# Supplementary figures and images for: Exploring the Action Mechanism of the Active Ingredient of Quercetin in Ligustrum lucidum on the Mouse Mastitis Model Based on Network Pharmacology and Molecular Biology Validation
Source: Evid Based Complement Alternat Med. 2022 Jun 10;2022:4236222. doi: 10.1155/2022/4236222 (PMC9205729; doi:10.1155/2022/4236222)

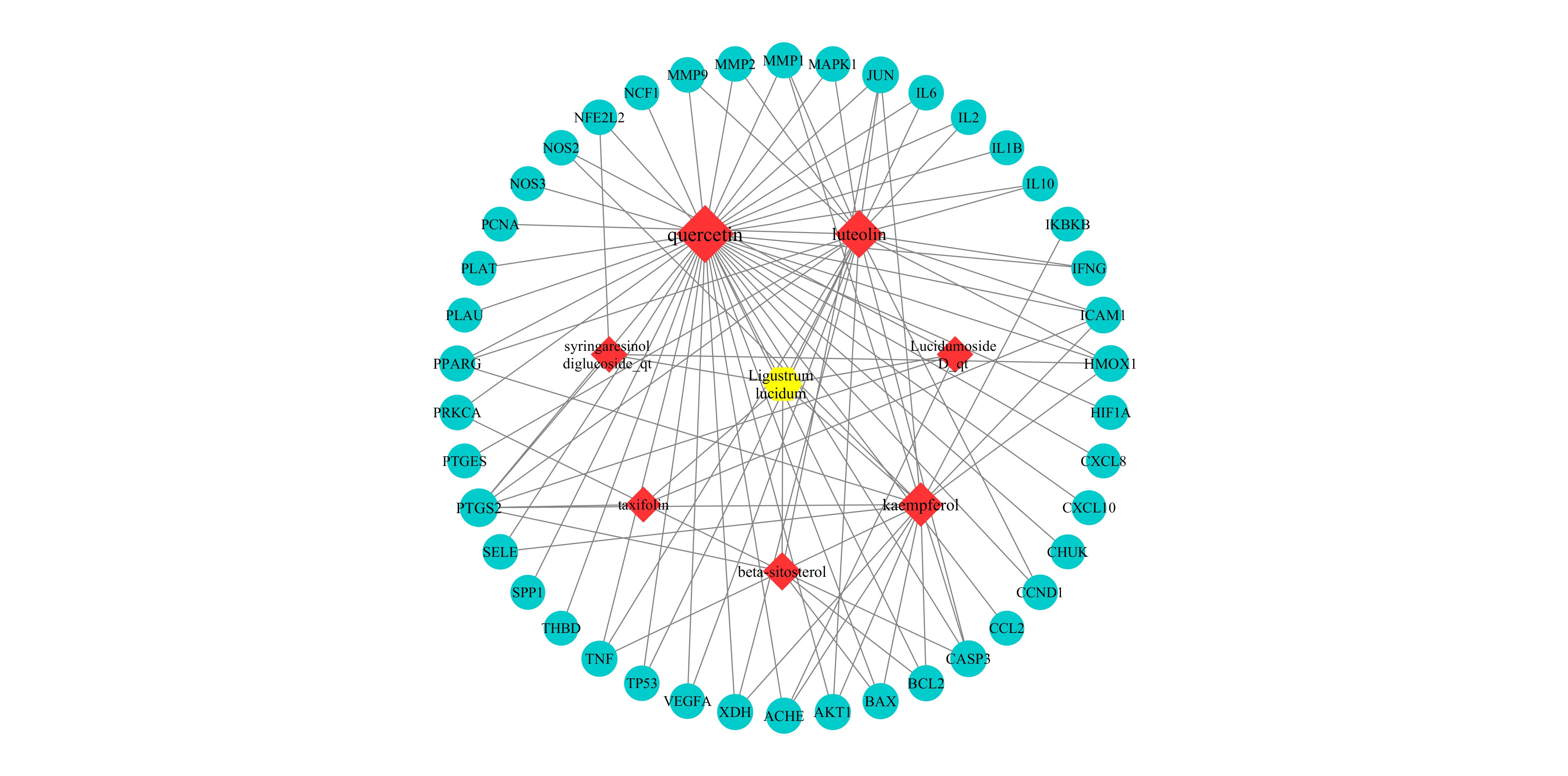

Supplement: Supplementary Materials — Figure 1: “TCM - active ingredient - Anti-inflammation target” network of forsythia. Figure 2: PPI network of common targets of forsythia and Anti-inflammation. Figure 3: Mouse breast tissues H&E staining(40 ×). Figure 4: Relative expression of TNF and IL6 mRNA at different time periods. Different lowercase letters indicate significant differences (P < 0.05); the same lowercase letters indicate not significant differences (P > 0.05); the same below. Figure 5: Expression of NF-κB, AKT1, TNF and IL6 proteins at different time periods. [file 4236222.f1.zip › 4236222.f1/Figure 1 (1).jpg]

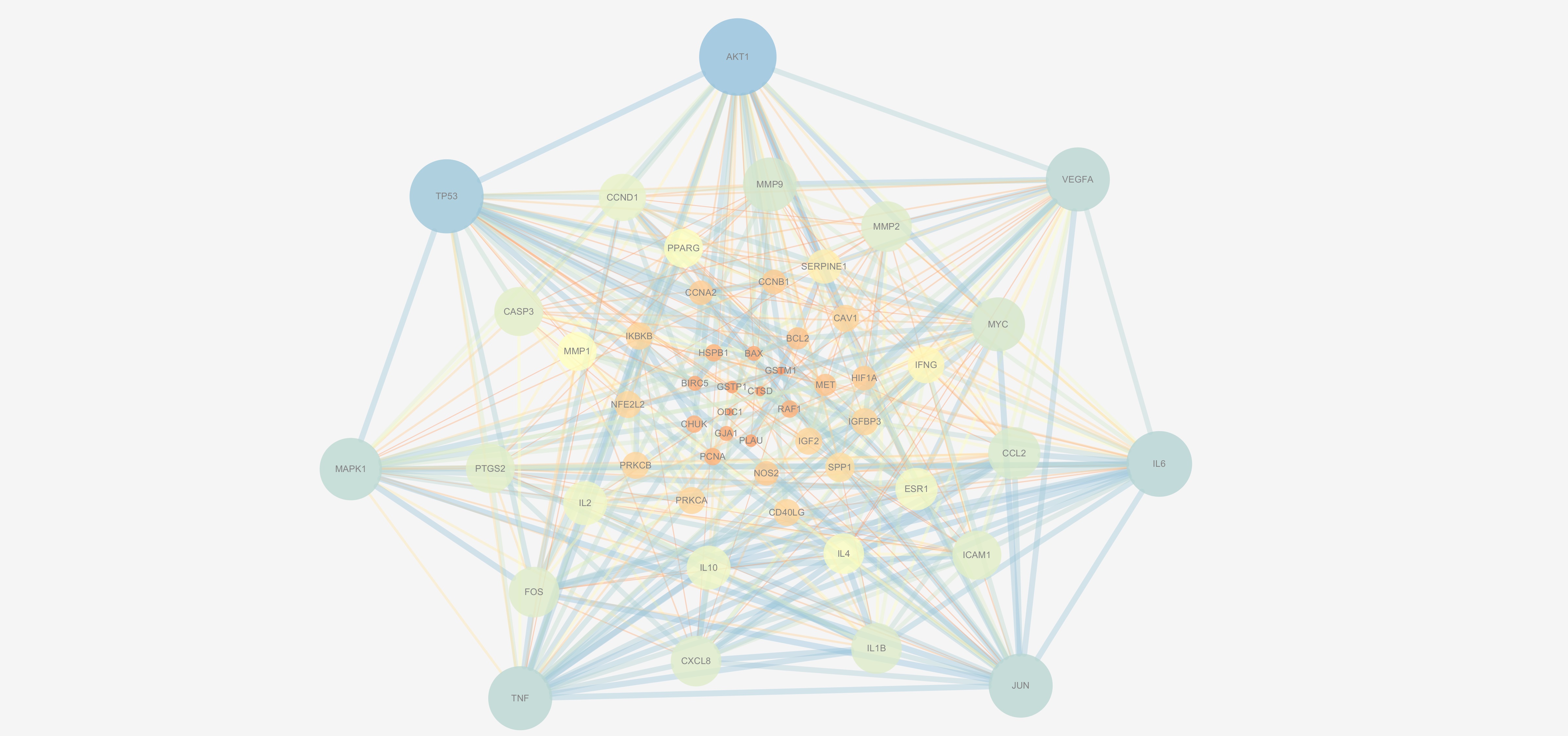

Supplement: Supplementary Materials — Figure 1: “TCM - active ingredient - Anti-inflammation target” network of forsythia. Figure 2: PPI network of common targets of forsythia and Anti-inflammation. Figure 3: Mouse breast tissues H&E staining(40 ×). Figure 4: Relative expression of TNF and IL6 mRNA at different time periods. Different lowercase letters indicate significant differences (P < 0.05); the same lowercase letters indicate not significant differences (P > 0.05); the same below. Figure 5: Expression of NF-κB, AKT1, TNF and IL6 proteins at different time periods. [file 4236222.f1.zip › 4236222.f1/Figure 2 (1).jpg]

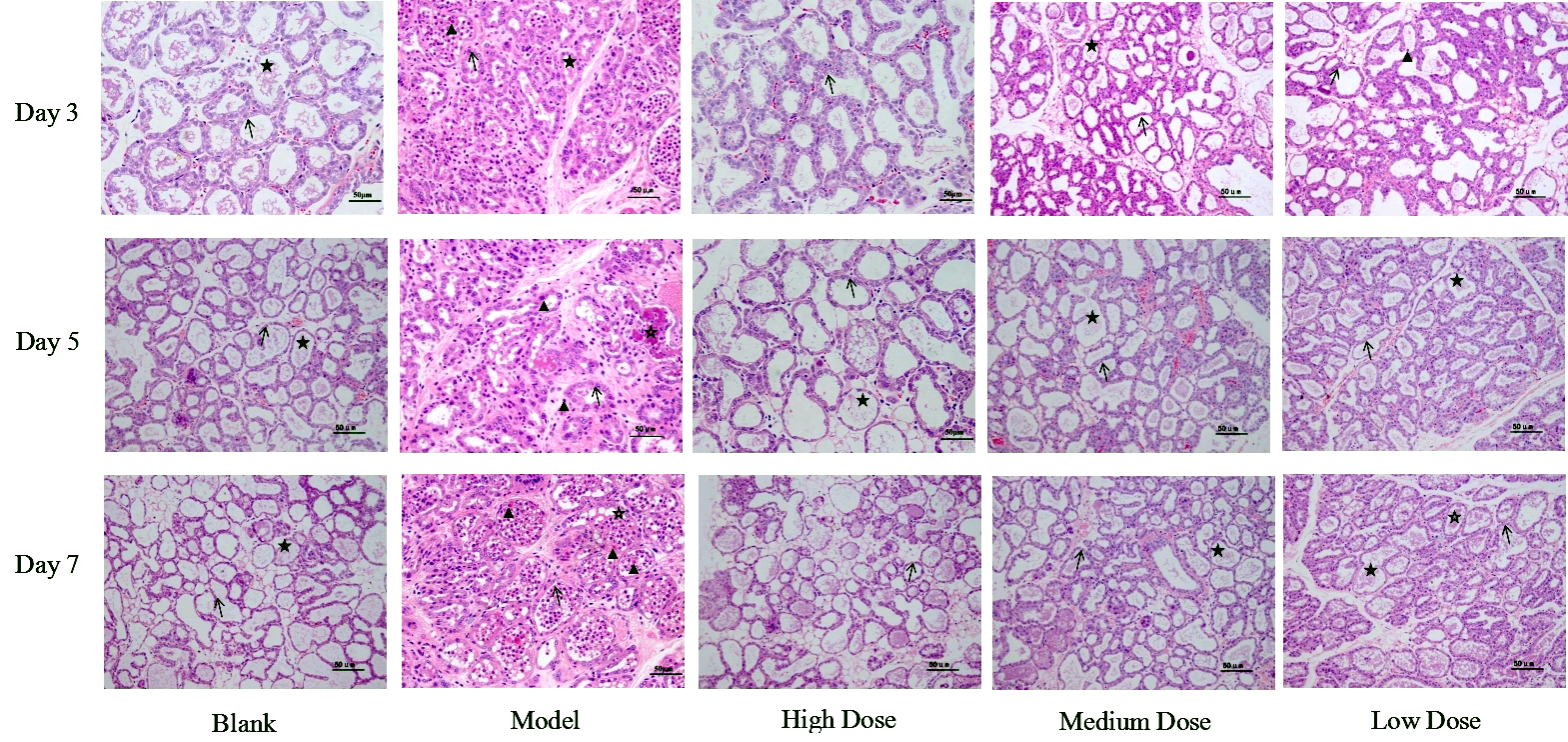

Supplement: Supplementary Materials — Figure 1: “TCM - active ingredient - Anti-inflammation target” network of forsythia. Figure 2: PPI network of common targets of forsythia and Anti-inflammation. Figure 3: Mouse breast tissues H&E staining(40 ×). Figure 4: Relative expression of TNF and IL6 mRNA at different time periods. Different lowercase letters indicate significant differences (P < 0.05); the same lowercase letters indicate not significant differences (P > 0.05); the same below. Figure 5: Expression of NF-κB, AKT1, TNF and IL6 proteins at different time periods. [file 4236222.f1.zip › 4236222.f1/Figure 3.tif]

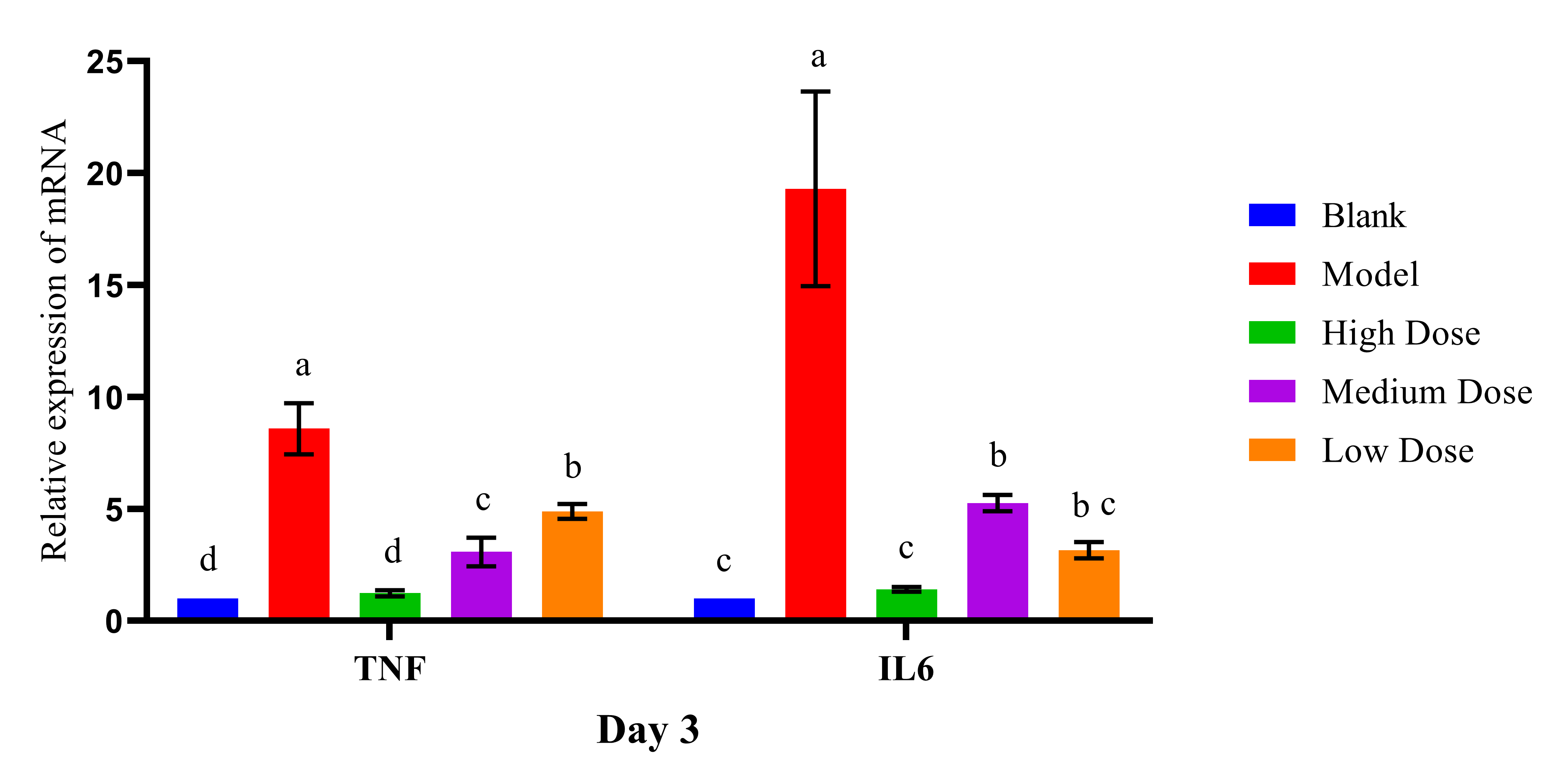

Supplement: Supplementary Materials — Figure 1: “TCM - active ingredient - Anti-inflammation target” network of forsythia. Figure 2: PPI network of common targets of forsythia and Anti-inflammation. Figure 3: Mouse breast tissues H&E staining(40 ×). Figure 4: Relative expression of TNF and IL6 mRNA at different time periods. Different lowercase letters indicate significant differences (P < 0.05); the same lowercase letters indicate not significant differences (P > 0.05); the same below. Figure 5: Expression of NF-κB, AKT1, TNF and IL6 proteins at different time periods. [file 4236222.f1.zip › 4236222.f1/Figure 4_Day3 qPCR.tif]

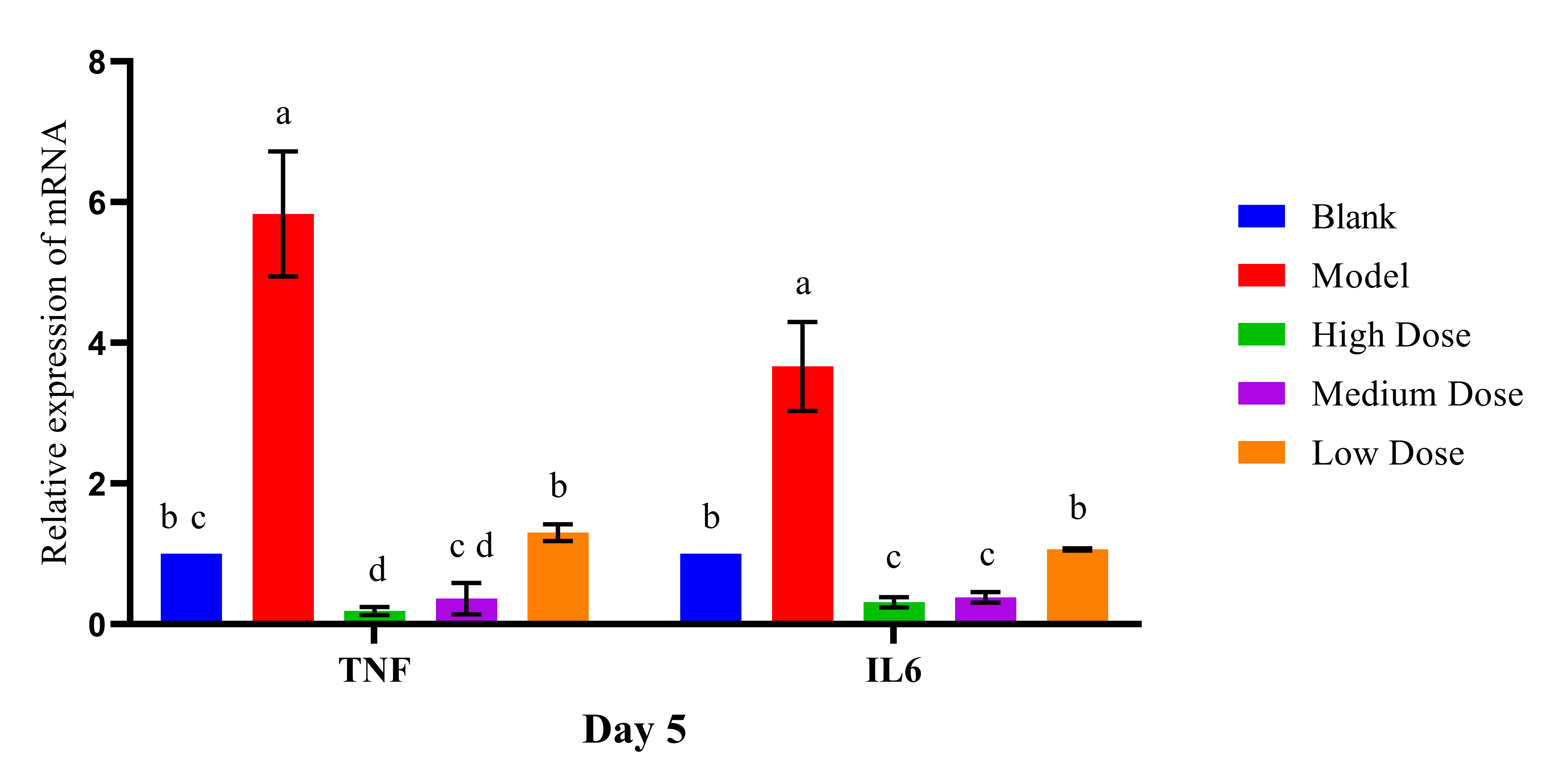

Supplement: Supplementary Materials — Figure 1: “TCM - active ingredient - Anti-inflammation target” network of forsythia. Figure 2: PPI network of common targets of forsythia and Anti-inflammation. Figure 3: Mouse breast tissues H&E staining(40 ×). Figure 4: Relative expression of TNF and IL6 mRNA at different time periods. Different lowercase letters indicate significant differences (P < 0.05); the same lowercase letters indicate not significant differences (P > 0.05); the same below. Figure 5: Expression of NF-κB, AKT1, TNF and IL6 proteins at different time periods. [file 4236222.f1.zip › 4236222.f1/Figure 4_Day5 qPCR.tif]

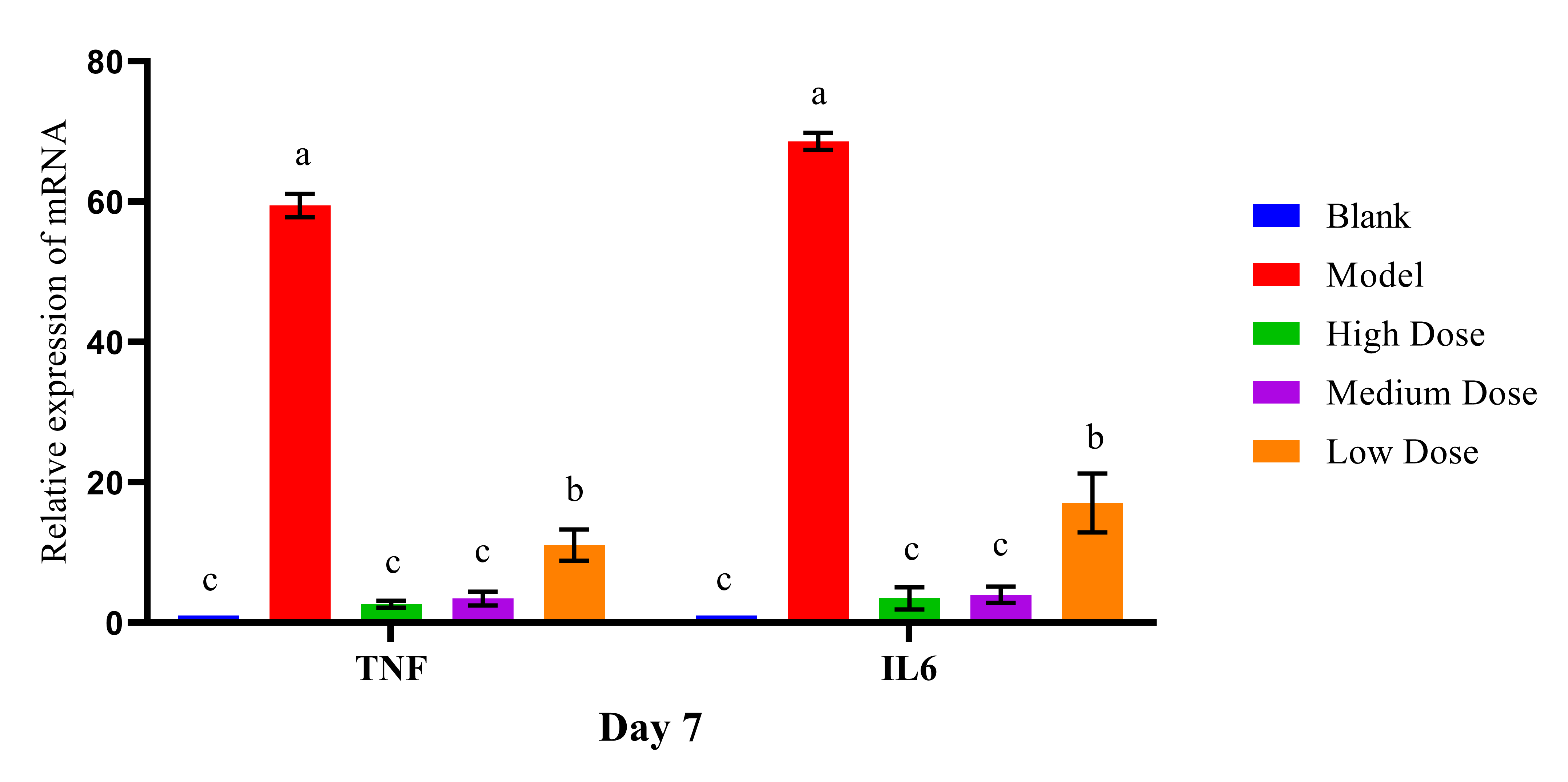

Supplement: Supplementary Materials — Figure 1: “TCM - active ingredient - Anti-inflammation target” network of forsythia. Figure 2: PPI network of common targets of forsythia and Anti-inflammation. Figure 3: Mouse breast tissues H&E staining(40 ×). Figure 4: Relative expression of TNF and IL6 mRNA at different time periods. Different lowercase letters indicate significant differences (P < 0.05); the same lowercase letters indicate not significant differences (P > 0.05); the same below. Figure 5: Expression of NF-κB, AKT1, TNF and IL6 proteins at different time periods. [file 4236222.f1.zip › 4236222.f1/Figure 4_Day7 qPCR.tif]

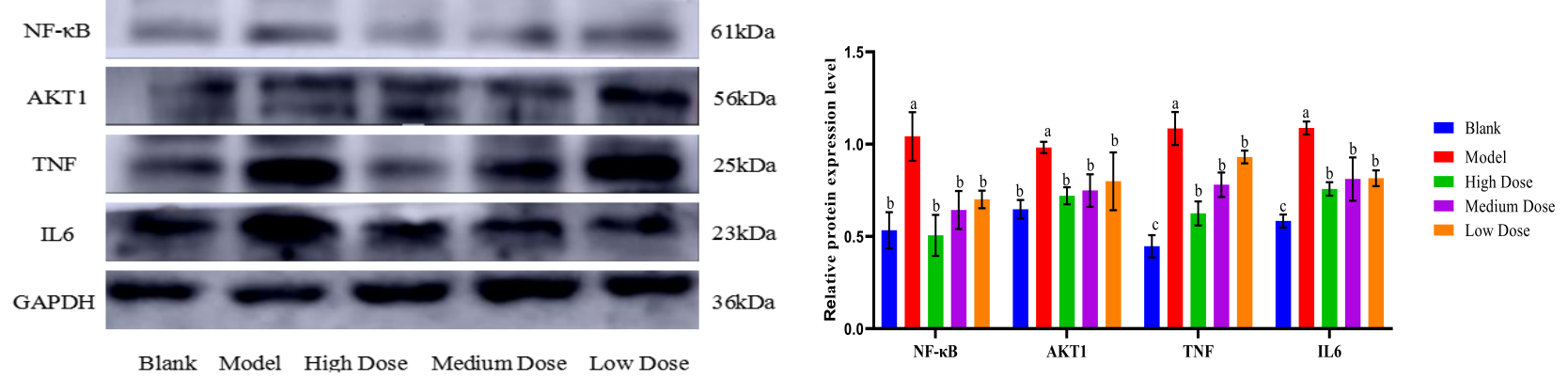

Supplement: Supplementary Materials — Figure 1: “TCM - active ingredient - Anti-inflammation target” network of forsythia. Figure 2: PPI network of common targets of forsythia and Anti-inflammation. Figure 3: Mouse breast tissues H&E staining(40 ×). Figure 4: Relative expression of TNF and IL6 mRNA at different time periods. Different lowercase letters indicate significant differences (P < 0.05); the same lowercase letters indicate not significant differences (P > 0.05); the same below. Figure 5: Expression of NF-κB, AKT1, TNF and IL6 proteins at different time periods. [file 4236222.f1.zip › 4236222.f1/Figure 5_Day3 WB.png]

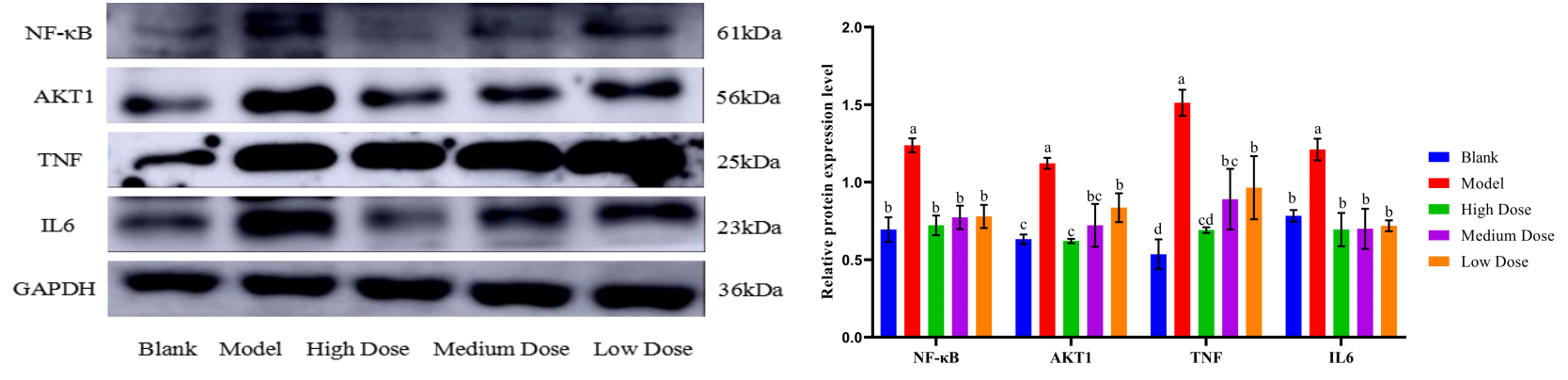

Supplement: Supplementary Materials — Figure 1: “TCM - active ingredient - Anti-inflammation target” network of forsythia. Figure 2: PPI network of common targets of forsythia and Anti-inflammation. Figure 3: Mouse breast tissues H&E staining(40 ×). Figure 4: Relative expression of TNF and IL6 mRNA at different time periods. Different lowercase letters indicate significant differences (P < 0.05); the same lowercase letters indicate not significant differences (P > 0.05); the same below. Figure 5: Expression of NF-κB, AKT1, TNF and IL6 proteins at different time periods. [file 4236222.f1.zip › 4236222.f1/Figure 5_Day5 WB.png]

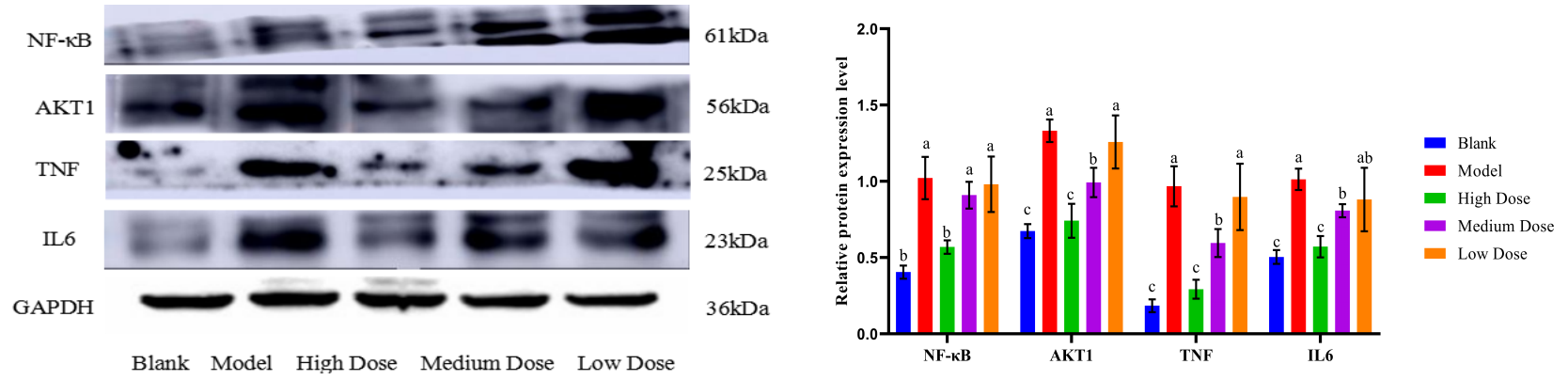

Supplement: Supplementary Materials — Figure 1: “TCM - active ingredient - Anti-inflammation target” network of forsythia. Figure 2: PPI network of common targets of forsythia and Anti-inflammation. Figure 3: Mouse breast tissues H&E staining(40 ×). Figure 4: Relative expression of TNF and IL6 mRNA at different time periods. Different lowercase letters indicate significant differences (P < 0.05); the same lowercase letters indicate not significant differences (P > 0.05); the same below. Figure 5: Expression of NF-κB, AKT1, TNF and IL6 proteins at different time periods. [file 4236222.f1.zip › 4236222.f1/Figure 5_Day7 WB.png]
